# Sympathetic modulation by antihypertensive drugs

**DOI:** 10.1111/jch.14334

**Published:** 2021-08-03

**Authors:** Kenichi Katsurada, Kazuomi Kario

**Affiliations:** ^1^ Division of Cardiovascular Medicine Department of Internal Medicine Jichi Medical University School of Medicine Shimotsuke Tochigi Japan; ^2^ Division of Clinical Pharmacology Department of Pharmacology Jichi Medical University School of Medicine Shimotsuke Tochigi Japan

The sympathetic nervous system (SNS) has been identified as a major contributor to the pathophysiology of hypertension. It is well known that dysregulations of the SNS, such as impairments of the baroreflex or exercise pressor reflex, are a common feature of hypertension. In a study published in this issue of the *Journal of Clinical Hypertension*,^1^ Peri‐Okonny and coworkers investigated the effects of eplerenone, a selective mineralocorticoid receptor (MR) antagonist, and amlodipine, a dihydropyridine calcium channel blocker, on exercise pressor reflex in hypertensive patients. The static handgrip exercise with post‐handgrip exercise circulatory arrest, which is an isometric exercise, was performed to assess metaboreflex, along with arm cycling, which is a dynamic exercise, to assess mechanoreflex. The results showed that eplerenone had no significant effects on metaboreflex and mechanoreflex in hypertensive patients; this is inconsistent with previous reports from another group including some of the same authors,^2^ which suggested that eplerenone had beneficial effects on exercise pressor reflex in animal models of hypertension. The authors proposed several factors that might account for this discrepancy, including (1) that the dose of eplerenone (average 119.6 mg) used in the current study was too low because it did not significantly lower blood pressure; (2) that the dose sufficient to induce central nervous system (CNS) effects by passing through the blood brain barrier in humans is unknown; and (3) that the potential action of eplerenone on sympathetic nerve activity may be evident only when the renin angiotensin system is activated. Mineralocorticoid receptors are widely expressed in the brain, kidney, and heart.^2^ It is of interest to note that MRs are also expressed in the spinal cord, dorsal root ganglia (DRG), and peripheral nerves, predominantly colocalized with calcitonin‐gene‐related peptide‐immunoreactive neurons,^3^ suggesting the possibility that mineralocorticoids interact with the afferent neural pathway to modulate sympathetic outflow via the CNS (Figure 1).

**FIGURE 1:**
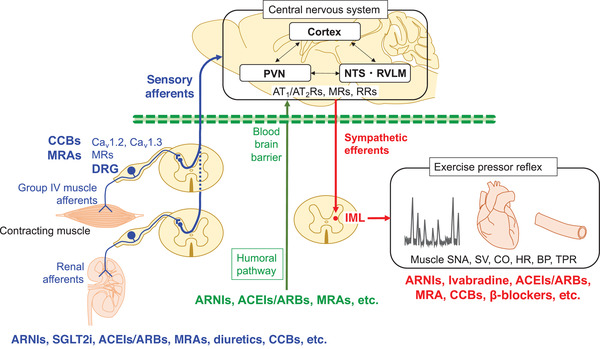
Proposed model for regulation of the exercise pressor reflex by the sympathetic nervous system and the potential sites targeted by antihypertensive drugs. *Abbreviations*: ACEI, angiotensin converting enzyme inhibitor; ARB, angiotensin receptor blocker; ARNI, angiotensin receptor neprilysin inhibitor; AT1/AT2Rs, angiotensin type 1/angiotensin type 2 receptors; BP, blood pressure; Ca_v_, voltage‐dependent L‐type calcium channel; CCB, calcium channel blocker; CO, cardiac output; DRG, dorsal root ganglia; HR, heart rate; IML, intermediolateral cell column; MRA, mineralocorticoid receptor antagonist; MRs, mineralocorticoid receptors; NTS, nucleus tractus solitarius; PVN, paraventricular nucleus; RRs, renin receptors; RVLM, rostral ventrolateral medulla; SGLT2i, sodium‐glucose cotransporter 2 inhibitor; SNA, sympathetic nerve activity; SV, stroke volume; TPR, total peripheral resistance

On the other hand, amlodipine was unexpectedly found to augment the metaboreflex, but not the mechanoreflex, in hypertensive patients.^1^ Amlodipine antagonizes four isoforms of the voltage‐dependent L‐type calcium channel, Ca_v_1.1, Ca_v_1.2, Ca_v_1.3, and Ca_v_1.4. Since Ca_v_1.2 and Ca_v_1.3 are expressed in the spinal cord and DRG, and implicated in the pathogenesis of neuropathic pain, amlodipine may block Ca_v_1.2 and Ca_v_1.3 to modulate the metaboreflex during static exercise.

Generally, peripheral factors, including reagents, send their information to the brain through two distinct pathways, the humoral and neural pathways (Figure 1). The humoral pathway includes the blood brain barrier. It has been reported that some of the antihypertensive drugs, such as angiotensin receptor neprilysin inhibitors (ARNIs),^4^ angiotensin converting enzyme inhibitors (ACEIs),^5^ angiotensin receptor blockers (ARBs),^6^ and MR antagonists (MRAs),^2^ can enter the brain through the blood brain barrier. The brain includes a discrete renin‐angiotensin system, that is, independent of circulatory renin angiotensin system. The angiotensin type 1 receptors (AT1Rs), angiotensin type 2 receptors (AT2Rs), MRs and renin receptors (RRs) are expressed in the brain, including in the cortex and cardiovascular centers, such as the paraventricular nucleus (PVN) in the hypothalamus and rostral ventrolateral medulla (RVLM) in the brain stem.^6^ The brain renin angiotensin system contributes to the regulation of sympathetic outflow. Activation of AT1Rs within the hypothalamus and the brain stem existing upstream of increased reactive oxygen species and decreased nitric oxide augments sympathetic outflow.^6^ Activation of the RRs producing Ang II in the brain increases blood pressure.^6^

The neural pathway is composed of the sympathetic afferent fibers innervating peripheral organs. Afferent signals are input to the dorsal horn at the spinal cord via the DRG, where the cell bodies of afferent fibers are located, and then conveyed to the CNS. The afferent arm of the metaboreflex is composed of the group IV muscle afferent fibers, the DRG and the brain (Figure 1). L‐type calcium channel blockers can act on the DRG, where Ca_v_1.2 and Ca_v_1.3 are expressed, to modulate the metaboreflex. Since MRs are expressed both in the DRG^3^ and in the brain,^2^ MRA can act on the DRG and/or brain to modulate the metaboreflex. In regard to calcium channel blockers, it has been reported that cilnidipine, which has an N‐type calcium channel blocking action, and azelnidipine have different effects from amlodipine. Cilnidipine was shown to suppress isometric exercise‐induced sympatho‐excitation, as assessed by pupillometry, more effectively than amlodipine.^7^ Cilnidipine also suppressed cardiac sympathetic overactivity assessed by ^123^I‐MIBG cardiac scintigraphy, while amlodipine exhibited little suppressive effect.^8^ Azelnidipine achieved significantly greater suppression of muscle sympathetic nerve activity, improvement of baroreflex sensitivity^9^ and suppression of exercise‐induced sympatho‐excitation assessed by pupillometry^10^ compared with amlodipine in hypertensive patients.

The kidney plays a critical role in regulating the SNS as well as the fluid balance and blood pressure. The kidneys have both efferent sympathetic and afferent sensory nerves. Afferent renal nerve signals are integrated at the level of the PVN, and their activation results in a general increase in sympathetic outflow.^11^, ^12^ Animal studies using rats with hypertension and heart failure have shown that resting afferent renal nerve activity is greater in these animals than in normal rats.^12^, ^13^ In the kidneys, AT1R/AT2R, MRs, and calcium channels are expressed in addition to the transporters related with diuresis and natriuresis. Many antihypertensive drugs, such as ACEIs/ARBs, MRAs, calcium channel blockers and diuretics, act on/target the kidneys.

In addition, it is possible that the new drugs for heart failure directly or indirectly act on the renal afferents to modulate the SNS. Sodium‐glucose cotransporter 2 (SGLT2) is localized in the proximal convoluted tubules of the kidney. SGLT2 inhibitors (SGLT2i) are widely used to treat heart failure with or without diabetes. SGLT2i also has antihypertensive effects^14^, ^15^, ^16^, ^17^ and sympatho‐inhibitory effects.^18^ Neprilysin is dominantly expressed in the kidney to degrade angiotensin II and natriuretic peptides. A new class of drugs, the ARNI is developed to treat heart failure and hypertension. ARNIs inhibit neprilysin activity in the kidney to increase circulating natriuretic peptides.

Renal denervation, a novel, innovative therapy for hypertension, is another approach to directly modulate the SNS. Renal denervation interrupts both the afferent inputs from the kidney to the CNS and the efferent outputs from the CNS to the kidney, resulting in suppression of sympathetic outflow and eliciting beneficial effects in both hypertension and heart failure.^12^, ^19^ It would be very worthwhile to assess whether the exaggerated muscle metaboreflex function in hypertension is affected by renal denervation.

The rise in blood pressure with the exercise pressor reflex is mainly due to increases in cardiac output (CO) and total peripheral resistance (TPR). CO is determined as the product of stroke volume and heart rate. TPR is defined as mean arterial pressure (MAP)/CO. All of these parameters are affected by drugs to treat hypertension and heart failure, including ARNIs, ivabradine, ACEIs/ARBs, MRAs, calcium channel blockers, and β‐blockers.

In conclusions, the current paper by Peri‐Okonny and coworkers^1^ demonstrated a potentially unfavorable potentiation of muscle metaboreflex function by amlodipine–versus no significant effect by eplerenone–in hypertensive patients. Overactivation of the SNS during exercise is one of the pathophysiological properties of hypertension and closely related with high risk of cardiovascular events. Cardiovascular regulation by the SNS is the process that integrates visceral inputs from various peripheral sites, including the muscle afferents and renal afferents within the CNS, to determine sympathetic outflow. Antihypertensive drugs potentially act on the various sites of this process to modulate sympathetic outflow (Figure 1). Further basic and clinical studies are needed to explore the potential targets and roles in regulating the SNS by each drug for hypertension and heart failure, and their synergistic effects.

## CONFLICT OF INTEREST

Kario K received research grant from MSD K.K., Astellas Pharma Inc., Eisai Co., Otsuka Pharmaceutical Co., Sanwa Kagaku Kenkyusho Co., Daiichi Sankyo Co., Taisho Pharmaceutical Co., Ltd, Sumitomo Dainippon Pharma Co., Takeda Pharmaceutical Co., Teijin Pharma, Boehringer Ingelheim Japan Inc., Bristol‐Myers Squibb K.K., Mochida Pharmaceutical Co., and honoraria from Daiichi Sankyo Co. Ltd, Mylan EPD outside the submitted work. The other author declare that they have no conflict of interest.
